# Why Will Not Fire Burn Brightly When the Sun Shines upon It?

**Published:** 1895-11

**Authors:** 


### Editorial, Etc.

WHY WILL NOT FIRE BURN BRIGHTLY WHEN THE SUN SHINES UPON IT?—This question naturally suggests itself whenever we attempt to light a match and watch its combustion in the full sunlight. A few moments' consideration of some of the principles which lie at the foundation of modern theories of combustion will enable us to answer this question intelligently.

What is flame? Answer—Flame is burning gas.

What is burning? Answer—Burning or combustion (as it occurs under ordinary conditions) is the rapid union of bodies with oxygen—a phenomen attended with the evolution of heat, and generally also with light. We may have combustion attended with intense heat, but with little light, as in the case of burning hydrogen, or with more or less heat and considerable light. as in the case of carbonaceous substances, as wood, oils, illuminating gas, etc.

The effect of sunlight upon chemical combinations is variable. With certain bodies, for example those employed in photographic processes, the action is rapid and powerful; in other instances, and in the one under consideration, sunlight is without appreciable effect. That is to say, in the combustion of wood, sunlight has no effect upon the rapidity of the intensity of the chemical action which we call burning.

It is true, however, that burning wood and other s ubstances undergoing combustion, appear far less brilliant in the sunlight than otherwise, but this is wholly the effect of contrast. The stars do not show themselves by daylight, and even the electric light—unless very intense—fails to cast a shadow upon a spot where the sun is also shining. So it is with the light from all burning bodies. The answer to our question then is this:

Fire only appears to burn less brightly in the sunshine than in the shade. The actual brilliancy and the rapidity of the Combustion are the same both in sunlight and in darknes. —*Popular Science News.*

